# Combination Therapy With Ensitrelvir and Remdesivir Versus Antiviral Monotherapy in Patients Receiving Anti-CD20 Monoclonal Antibody Therapy: A Retrospective Cohort Study

**DOI:** 10.7759/cureus.99402

**Published:** 2025-12-16

**Authors:** Tomonori Takano, Hiroyuki Aiba, Hiromu Takemura, Hiroyuki Kunishima

**Affiliations:** 1 Department of Infectious Diseases, St. Marianna University School of Medicine, Kawasaki, JPN; 2 Department of Pediatrics, St. Marianna University School of Medicine, Kawasaki, JPN; 3 Department of Microbiology, St. Marianna University School of Medicine, Kawasaki, JPN

**Keywords:** anti-cd20 antibody therapy, combination therapy, covid-19, ensitrelvir, remdesivir

## Abstract

Background

We aimed to evaluate the effectiveness of combination therapy with ensitrelvir and remdesivir compared to antiviral monotherapy in patients with a history of anti-CD20 monoclonal antibody therapy who were hospitalized with COVID-19.

Methods

This retrospective cohort study was conducted at St. Marianna University Hospital (a tertiary care hospital) in Kawasaki, Kanagawa, Japan. Among 1,549 patients hospitalized with COVID-19 between April 2022 and December 2024, 17 patients with a history of anti-CD20 monoclonal antibody therapy were included in the study. Among them, seven patients received combination therapy (ensitrelvir and remdesivir), and 10 received antiviral monotherapy. Ensitrelvir and remdesivir were administered for five and five to 10 days, respectively.

Results

No deaths occurred within 30 days. The one-year mortality rate was significantly lower in the combination group (1/7, 14.3%) than in the monotherapy group (7/9, 77.8%; p = 0.041). Case 8 was excluded from this analysis due to insufficient follow-up duration. At baseline, the combination therapy group had a higher proportion of patients with severe or critical COVID-19 (6/7, 85.7% vs. 2/10, 20.0%), a lower median serum IgG level (526 vs. 908.5 mg/dL), and a higher prevalence of pneumonia (6/7, 85.7% vs. 2/10, 40.0%). Despite these more severe baseline characteristics, the combination therapy group showed significantly lower post-treatment antigen levels (median (IQR): 11.24 pg/mL (1.76-49.39 pg/mL) vs. 2792.41 pg/mL (186.9-5000 pg/mL), p = 0.0303).

Conclusions

Combination therapy with ensitrelvir and remdesivir was associated with lower long-term mortality and viral burden in patients with COVID-19 who had received anti-CD20 monoclonal antibody therapy. However, these results are based on a small retrospective cohort and should be interpreted as hypothesis-generating; larger prospective studies are needed to determine whether this association reflects a true therapeutic effect.

## Introduction

Since the report of the first case of COVID-19 in Wuhan, China, in late 2019, there have been changes in the pathogenicity and immune evasion capability of SARS-CoV-2 [1]. The in-hospital mortality rates of patients with COVID-19 during the alpha, delta, and omicron variant periods were 10%, 9%, and 6%, respectively, indicating a gradual reduction in viral pathogenicity [2]. However, the mortality rate of patients with COVID-19 who had a history of anti-CD20 antibody therapy was 14.7% [3]. The prolonged inflammation in these patients could be attributed to impaired viral clearance resulting from B-cell depletion [4-6]; however, the exact mechanism remains unclear.

Patients receiving anti-CD20 antibody therapy exhibit prolonged viral shedding and persistent infections [7]. Furthermore, the prolonged viral shedding in these patients is associated with an increased risk of onward transmission [8]. Accordingly, early reduction of the viral load is crucial in these patients. However, even with antiviral monotherapy or a combination of monoclonal antibodies against SARS-CoV-2 and antiviral agents, there are limitations to achieving effective viral clearance [9,10]. Recently, the administration of two antiviral agents with different mechanisms of action has been suggested to improve the clinical outcomes in patients with COVID-19 [11,12]. In the present study, we aimed to investigate short- and long-term clinical outcomes, as well as post-treatment changes in viral load, in patients with a history of anti-CD20 antibody therapy who received antiviral monotherapy or combination therapy with ensitrelvir and remdesivir. The primary objective was to compare one-year mortality. Secondary outcomes included 30-day mortality and SARS-CoV-2 antigen reduction.

## Materials and methods

Study design and participants

This retrospective observational study was conducted in accordance with the Strengthening the Reporting of Observational Studies in Epidemiology (STROBE) guidelines [13]. The study involved patients with a history of anti-CD20 monoclonal antibody therapy who were hospitalized with COVID-19 between April 2022 and December 2024 at St. Marianna University Hospital, a tertiary care center located in Kawasaki, Kanagawa, Japan.

During the study period, 1,549 patients were hospitalized for COVID-19. Among them, 17 patients who had received anti-CD20 monoclonal antibody therapy were included in the study.

**Figure 1:**
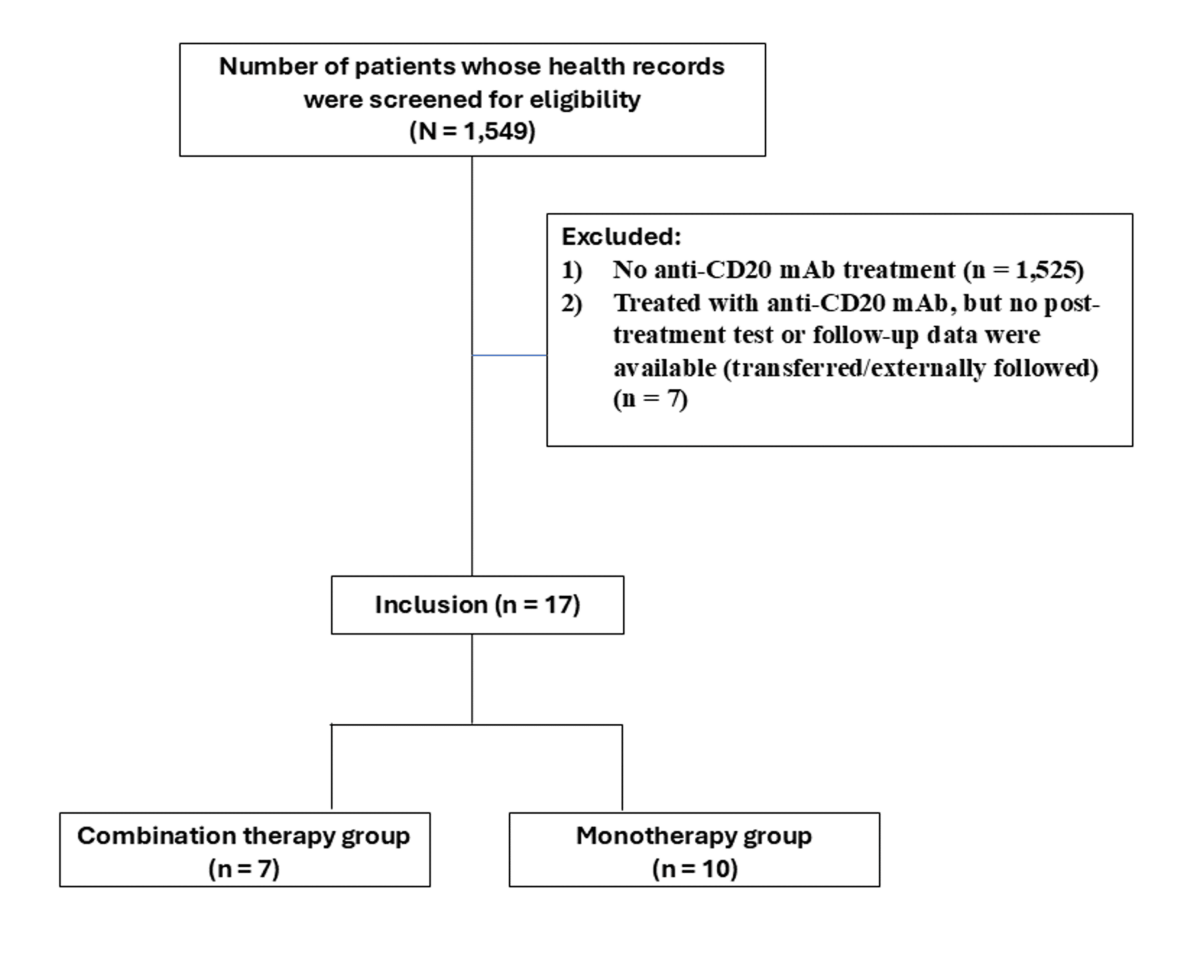
Flowchart of participant selection based on the inclusion and exclusion criteria mAb: monoclonal antibody

We excluded patients without pre- or post-treatment SARS-CoV-2 testing and those whose post-treatment clinical course was unavailable due to transfer or follow-up at another institution. Based on these criteria, seven patients were excluded. Figure 1 summarizes the inclusion and exclusion criteria.

Testing and treatment

Regarding the detection of SARS-CoV-2, nucleic acid amplification was performed using GeneXpert (Cepheid, Sunnyvale, CA, USA), BD MAX (Becton Dickinson and Company, Franklin Lakes, NJ, USA), and Cobas Liat (Roche Diagnostics, Rotkreuz, Switzerland). Antigen quantification was conducted using LUMIPULSE® SARS-CoV-2 Ag (Fujirebio Inc., Tokyo, Japan) and HISCL™ SARS-CoV-2 Ag (Sysmex Corporation, Kobe, Japan). The LUMIPULSE® assay has a quantifiable range of 0.60-5,000 pg/mL. Samples with values <1.0 pg/mL and >10 pg/mL were considered negative and positive, respectively [14]. For the HISCL assay, samples with values ≥3.65 pg/mL were considered positive [15]. The date of diagnosis was defined as day 0 of the illness, which corresponded to the onset of symptoms. For asymptomatic cases, the date of the first positive test result was considered day 0.

In this study, the antiviral agents included molnupiravir (Merck & Co., Inc., Rahway, NJ, USA), nirmatrelvir/ritonavir (Pfizer Inc., New York City, NY, USA), ensitrelvir (Shionogi & Co., Ltd., Osaka, Japan), and remdesivir (Gilead Sciences Inc., Foster City, CA, USA). The antiviral agents were administered based on the recommendations provided in the respective prescription information. The choice between combination therapy and monotherapy was determined at the discretion of the treating physician based on clinical judgment. For combination therapy, patients were treated with ensitrelvir (375 mg orally once on day 1, followed by 125 mg orally once daily from days 2 to 5) and remdesivir (200 mg intravenously on day 1, followed by 100 mg intravenously once daily from day 2 for up to 10 days). Table 1 shows the durations of antiviral agent administration.

**Table 1:** Details of individual cases and antiviral regimens Severity was classified according to the World Health Organization (WHO) COVID-19 severity scale [16].*The Charlson Comorbidity Index was calculated using a previously described methodology [17]. **Only Case 8 did not reach one year after COVID-19 onset at the time of retrospective observation, having elapsed 283 days since infection.

	Case	Age (years)/sex	Comorbidity	Pneumonia on chest imaging	Severity	Mechanical ventilation required	Serum IgG (mg/dL)	Days from last anti-CD20 antibody administration to onset	Charlson comorbidity index*	Antiviral agents and duration (days)	Days from onset to death
Combination therapy	1	71/female	Follicular lymphoma	Present	Severe	No	343	Obinutuzumab, 41	6	Remdesivir and ensitrelvir (both for 5 days)	Alive
	2	52/male	Diffuse large B-cell lymphoma	Absent	Moderate	No	664	Rituximab, 12	4	Remdesivir and ensitrelvir (both for 5 days)	Alive
	3	81/male	Follicular lymphoma	Present	Severe	No	832	Rituximab, 170	8	Molnupiravir (5 days), then remdesivir and ensitrelvir (both for 5 days)	Alive
	4	67/male	Primary macroglobulinemia	Present	Severe	No	518	Rituximab, 5	7	Remdesivir (10 days) and ensitrelvir (5 days)	Alive
	5	77/female	Nodal marginal zone B cell lymphoma	Present	Severe	No	428	Rituximab, 554	6	Remdesivir and ensitrelvir (both for 5 days)	Alive
	6	68/male	Nephrotic syndrome	Present	Severe	No	526	Rituximab, 44	6	Remdesivir and ensitrelvir (both for 5 days)	Alive
	7	53/male	Diffuse large B-cell lymphoma	Present	Critical	Yes	554	Rituximab, 213	4	Remdesivir and ensitrelvir (both for 5 days)	57
Monotherapy	8	20/female	Systemic lupus erythematosus	Absent	Mild	No	945	Rituximab, 4	1	Remdesivir (5 days)	Alive**
	9	69/female	Relapsed primary central nervous system lymphoma	Absent	Moderate	No	1530	Rituximab, 3887	6	Remdesivir (5 days)	78
	10	39/male	Dermatomyositis	Absent	Moderate	No	246	Rituximab, 289	2	Remdesivir (5 days)	341
	11	66/female	Follicular lymphoma	Present	Severe	No	417	Obinutuzumab, 34	5	Molnupiravir (5 days) and then remdesivir (5 days)	Alive
	12	78/female	Diffuse large B-cell lymphoma	Absent	Moderate	No	351	Rituximab, 37	7	Remdesivir (5 days)	35
	13	93/male	Diffuse large B-cell lymphoma	Present	Moderate	No	872	Rituximab, 20	7	Remdesivir (5 days)	42
	14	43/male	Diffuse large B-cell lymphoma	Absent	Mild	No	1389	Rituximab, 5	3	Remdesivir (5 days)	Alive
	15	68/male	Diffuse large B-cell lymphoma	Present	Moderate	No	1005	Rituximab, 109	6	Remdesivir (5 days)	62
	16	91/female	Diffuse large B-cell lymphoma	Absent	Mild	No	1125	Rituximab, 4	10	Remdesivir (5 days)	216
	17	59/female	Nodal marginal zone lymphoma	Present	Critical	Yes	142	Obinutuzumab, 188	4	Remdesivir (10 days)	129

In both groups, ensitrelvir was administered for five days.

Study outcomes

The primary outcomes were the 30-day and one-year mortality rates. Patients without ≥365 days of follow-up were excluded from the one-year mortality analysis. For fatal cases, additional information was collected to identify the cause of death and determine whether it occurred after the clinical improvement of COVID-19 symptoms. The secondary outcome was the between-group comparison of post-treatment SARS-CoV-2 antigen levels, which were defined as values obtained using the LUMIPULSE® assay on the date closest to the end of the antiviral therapy. Additionally, we performed between-group comparisons of patient background characteristics, including pre-treatment serum IgG levels, presence of pneumonia, disease severity, Charlson Comorbidity Index scores [17], and requirement for mechanical ventilation. Disease severity was classified based on the World Health Organization criteria [16].

Statistical analysis

Continuous variables are reported as mean with standard deviation or median with interquartile range (IQR); categorical variables are expressed as frequency with percentage. Given the small cohort size, between-group comparisons of categorical variables were performed using Fisher’s exact test. The Wilcoxon rank-sum test was used for between-group comparisons of non-normally distributed continuous variables. Additionally, conditional logistic regression was performed to estimate the odds ratios and attributable fractions for mortality. Survival curves were estimated using the Kaplan-Meier method and compared between the groups using the log-rank test. Antigen levels <0.6 or >5000 pg/mL were approximated and analyzed using the earliest post-treatment Lumipulse result. Statistical significance was set at a two-sided p-value of <0.05. All statistical analyses were performed using Stata version 16 (StataCorp, College Station, TX, USA).

## Results

Patient characteristics and comorbidities

Among the 17 included patients, seven received combination therapy and 10 received monotherapy. The most common comorbidity indicating the need for anti-CD20 monoclonal antibody therapy was hematological malignancy (85.7% and 80.0% in the combination therapy and monotherapy groups, respectively). There was no significant between-group difference in the Charlson Comorbidity Index (6.0 (5.0-6.5) and 5.5 (3.25-6.75) for the combination therapy and monotherapy groups, respectively; p = 0.487). The median serum IgG level was higher in the monotherapy group than in the combination therapy group, but not significantly (908.5 mg/dL vs. 526 mg/dL; p = 0.380). Furthermore, the incidence of pneumonia was higher in the combination therapy group than in the monotherapy group, but not significantly (6/7 vs. 4/10; p = 0.082). Similarly, one patient in each group required mechanical ventilation, with no significant between-group difference (p = 1.00). The combination therapy group had a significantly higher proportion of severe or critical cases than the monotherapy group (6/7 (85.7%) vs. 2/10 (20.0%); p = 0.015; Table 2).

**Table 2:** Patient characteristics IQR: interquartile range. Values are expressed as median (interquartile range) or number (%), as appropriate. Continuous variables were compared using the Wilcoxon rank-sum test (Mann–Whitney U test), and categorical variables using Fisher’s exact test. Severity was classified according to the World Health Organization COVID-19 severity scale [16].

		Combination therapy (7 cases）	Monotherapy (10 cases）	p-value
	Median age (IQR, years)	68 (60.0, 74.0)	67 (47.0, 75.75)	0.77
	Sex (Female ,N(%))	2, 28.6%	6, 60%	0.622
Comorbidity	Hematologic malignancy	6	8	
	Systemic lupus erythematosus	0	1	
	Dermatomyositis	0	1	
	Nephrotic syndrome	1	0	
	Charlson comorbidity index (IQR)	6.0 (5.0–6.5)	5.5 (3.25–6.75)	0.519
	Serum IgG value (IQR）	526 mg/dL (473.0, 609.0)	908.5 mg/dL (367.0, 1095.0)	0.417
	Pneumonia on chest imaging (N(%))	6 (85.7%)	4 (40.0%)	0.134
	Mechanical ventilation required	1 (14.3％)	1 (10.0%)	1
Severity	Non-sever (N(%))	1 (14.3％)	8 (80.0%)	0.015
	Severe (N(%))	5 (71.4%)	1 (10.0%)	
	Critical (N(%))	1 (14.3%)	1 (10.0%)	

Table 1 provides details regarding the individual patient cases, including the specific antiviral agents used, treatment duration, and survival status at one year after COVID-19 onset.

Primary outcome

No deaths occurred within 30 days of COVID-19 onset. The one-year mortality rate was significantly lower in the combination therapy group (14.3%) than in the monotherapy group (77.8%; p = 0.041). Conditional logistic regression revealed that the odds of death were 21 times higher in the monotherapy group than in the combination therapy group (OR 21.0, 95% CI: 1.09-1098.97, p = 0.012; Table 3).

**Table 3:** Conditional logistic regression analysis for one-year mortality *Reference category: monotherapy. Conditional logistic regression was performed to evaluate the association between treatment modality and one-year mortality.

	Odds Ratio	95% CI	p-value
Combination therapy vs. monotherapy	21	1.09–1098.97	0.012

**Figure 2:**
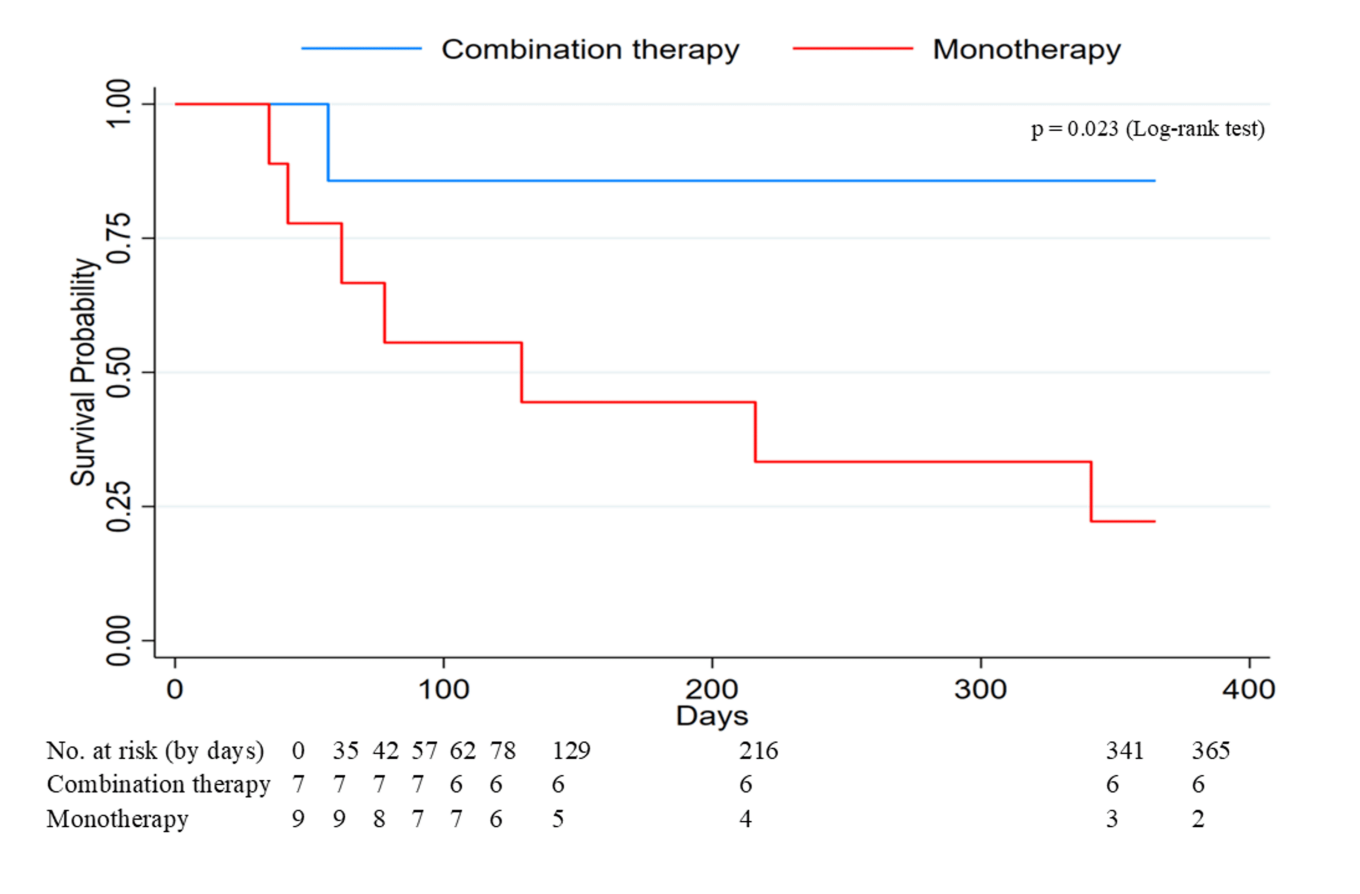
Kaplan–Meier survival curves for one-year mortality in patients receiving combination therapy versus monotherapy Survival probability was estimated using the Kaplan–Meier method. Blue line, combination therapy (ensitrelvir + remdesivir); red line, antiviral monotherapy. Time 0 was the date of symptom onset (or the first positive test for asymptomatic cases). Patients alive at 365 days were censored at day 365. The number of patients at risk is shown below the x-axis at prespecified time points (0, 35, 42, 57, 62, 78, 129, 216, 341, and 365 days).

The Kaplan-Meier analysis further demonstrated a significant survival difference between the groups (log-rank p = 0.023; Figure 2).

Cases 7 and 17 in the combination therapy and monotherapy groups, respectively, died without clinical improvement of COVID-19 (Table 4).

**Table 4:** Age, sex, underlying conditions, and causes of death in the eight fatal cases ARDS: acute respiratory distress syndrome; VAP: ventilator-associated pneumonia

Case	Age (years)/sex	Comorbidity	Cause of death
7	53/male	Diffuse large B-cell lymphoma	ARDS secondary to COVID-19
9	69/female	Relapsed primary central nervous system lymphoma	Brainstem compression secondary to malignant lymphoma following recovery from COVID-19
10	39/male	Dermatomyositis	Exacerbation of heart failure after COVID-19 recovery
12	78/female	Diffuse large B-cell lymphoma	Death in care facility after COVID-19 recovery; cause unknown
13	93/male	Diffuse large B-cell lymphoma	Death at home after recovery from COVID-19; cause unknown
15	68/male	Diffuse large B-cell lymphoma	Febrile neutropenia and candidemia after recovery from COVID-19
16	91/female	Diffuse large B-cell lymphoma	Death due to frailty following COVID-19 recovery and reduced food intake
17	59/female	Nodal marginal zone lymphoma	VAP following prolonged ventilatory support after COVID-19

The remaining six deaths occurred after improvement of the COVID-19 symptoms.

Secondary outcomes

LUMIPULSE® antigen testing was performed in six and five patients in the combination therapy and monotherapy groups, respectively. Table 5 summarizes the SARS-CoV-2 test results during hospitalization. The median number of days from symptom onset to initiation of antiviral therapy was 13 (IQR: 4.5-16.0) days in the combination therapy group and 0 (IQR: 0.0-3.0) days in the monotherapy group.

**Table 5:** Results of antigen quantification and nucleic acid amplification testing during hospitalization for each case GX: GeneXpert®; BD: BD MAX™; LI: Cobas® Liat®; LM: LUMIPULSE® SARS-CoV-2 Ag; HS: HISCL™ SARS-CoV-2 Ag; FA: fluorescent antigen; NA: nucleic acid Antigen levels are expressed in pg/mL. The number of days from symptom onset to treatment completion was calculated using each respective date as day 0.

Case	Days from symptom onset to initiation of antiviral therapy	Pre-treatment test results and days from symptom onset	First post-treatment test results and days elapsed since the end of antiviral therapy	Second post-treatment test results and days elapsed since the end of antiviral therapy	Third post-treatment test results and days elapsed since the end of antiviral therapy	Fourth post-treatment test results and days elapsed since the end of antiviral therapy
1	2	GX: 27.7, day 0	LM: <0.6, day 10	-	-	-
2	0	LM: >5000, day 0	LM: 19.5, day 10	LM: <0.6, day 14	-	-
3	19	LM: >5000, day 19	LM: 50.74, day 6	-	-	-
4	49	GX: 25.5, day 49	GX: 38.2, day 6	-	-	-
5	13	LM: >5000, day 13	LM: 2.98, day 5	-	-	-
6	13	LI: 21.1, day 13	LM: 1.35, day 10	-	-	-
7	7	LM: >5000, day 7	LM: 165.51, day 5	LM: 647.68, day 8	LM: 1055.8, day 16	-
8	3	FA: NA, day 2	LM: 2792.41, day 5	LM: 0.64, day 14	-	-
9	3	GX: 18.2, day 3	LM: 186.9, day 5	LM: 589.77, day 14	-	-
10	3	LM: >5000, day 3	LM: 3.24, day 10	-	-	-
11	8	HS: 172, day 0	HS: 2350, day 20	HS: 35.8, day 30	HS: <0.1, day 58	-
12	0	HS: 5576, day 0	HS: 37543.6, day 24	-	-	-
13	0	BD: NA, day 0	HS: 38671.4, day 11	HS: 1771.8, day 15	HS: 13.1, day 20	-
14	0	LM: >5000, day 0	HS: 559, day 20	HS: 2.5, day 45	HS: 1, day 1	-
15	0	HS: 29507.8, day 0	LM: >5000, day 10	-	-	-
16	0	LM: >5000, day 0	HS: 20192.4, day 15	HS: 85, day 21	HS: 828.9, day 23	-
17	0	GX: 29.6, day 0	GX: 18.9, day 15	GX: 20.1, day 22	LM: >5000, day 27	GX: 21.4, day 33

**Figure 3:**
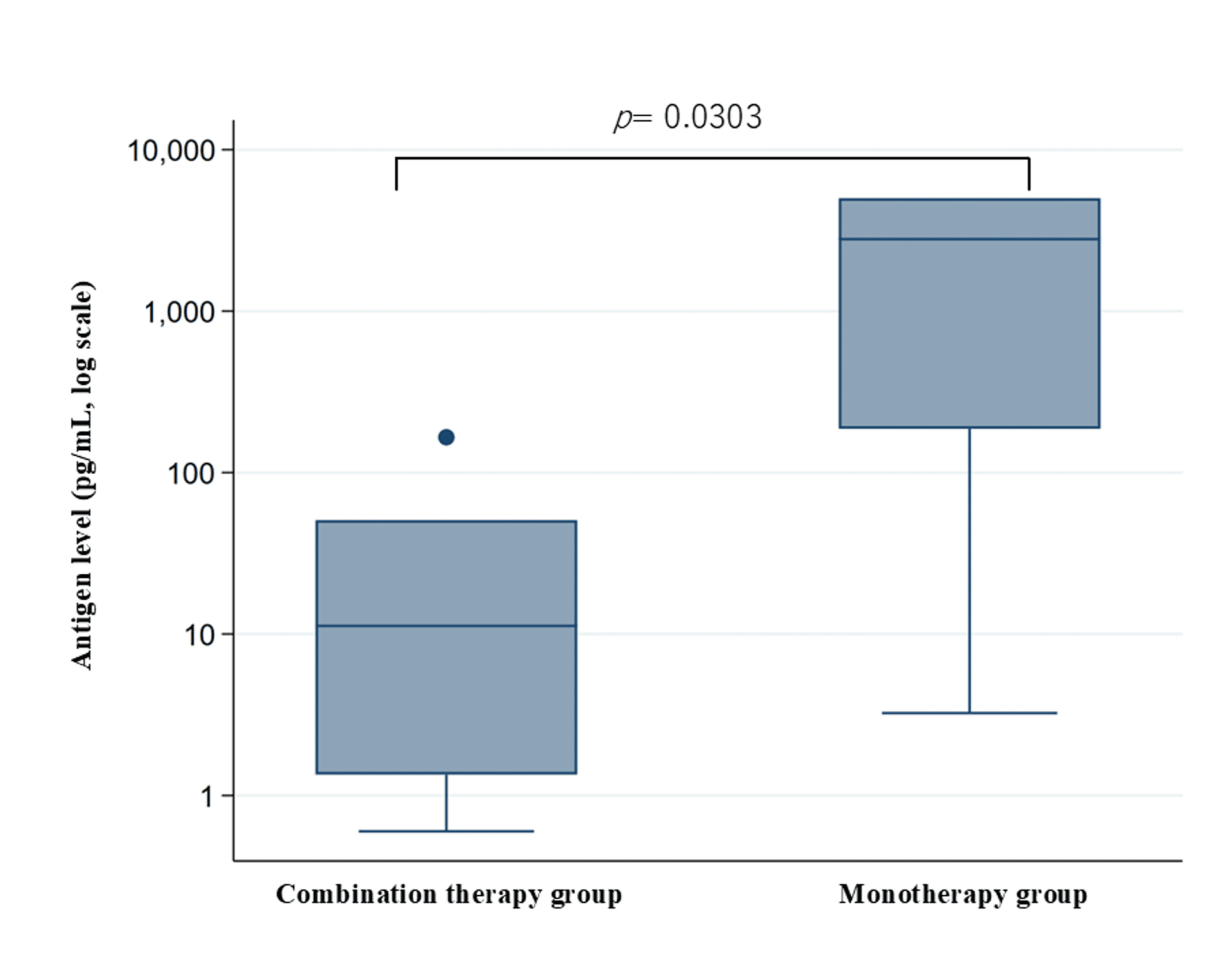
Box-and-whisker plots of post-treatment SARS-CoV-2 antigen levels in the combination and monotherapy groups Each box shows the interquartile range (IQR: 25^th^ to 75^th^ percentile), with the horizontal line inside the box indicating the median value. Whiskers extend to the smallest and largest values within 1.5 times the IQR from the lower and upper quartiles, respectively. Values outside this range are plotted individually as dots, and they represent statistical outliers. The y-axis is presented on a logarithmic scale (base 10) to accommodate the wide dynamic range of antigen concentrations. For values reported as below the detection limit (<0.6 pg/mL) or above the upper limit (>5000 pg/mL), 0.6 and 5000 pg/mL were used as approximations for analysis. Between-group comparisons were performed using the Wilcoxon rank-sum test.

The post-treatment SARS-CoV-2 antigen levels were visualized using box plots on a logarithmic scale (Figure 3).

The median number of days from treatment initiation to antigen testing was shorter in the combination therapy group (7.0 (IQR: 5.25-9.5) days) than in the monotherapy group (10.0 (IQR: 7.0-10.0) days). Furthermore, the median post-treatment antigen level was significantly lower in the combination therapy group than in the monotherapy group (11.24 pg/mL (IQR: 1.76-49.39 pg/mL) vs. 2792.41 pg/mL (IQR: 186.9-5000 pg/mL), respectively; p = 0.0303), which suggested that combination therapy achieved more effective reduction in viral antigen level.

## Discussion

Our findings indicated that the combination therapy group had a significantly lower one-year mortality rate and post-treatment SARS-CoV-2 antigen levels than the monotherapy group. Notably, these findings were observed despite the more severe disease profiles of patients in the combination therapy group than those in the monotherapy group, including lower serum IgG levels, a higher incidence of pneumonia, and a significantly greater proportion of severe or critical COVID-19 cases.

COVID-19 causes prolonged dysfunction in multiple organ systems [18]. In immunocompromised patients, persistent viral infection can lead to the progressive deterioration of multiple organ functions [4-7]. Accordingly, the relatively low mortality rate in the combination therapy group may be attributed to the significant post-treatment reduction in viral antigen levels. Notably, six of the seven deaths in the monotherapy group occurred after clinical improvement of the COVID-19 symptoms. Post-treatment SARS-CoV-2 antigen levels remained higher in the monotherapy group than in the combination group. This finding suggests that persistent viral infection may have contributed to progressive multi-organ dysfunction and eventual death. This phenomenon is especially relevant in patients with a history of anti-CD20 monoclonal antibody therapy, who are known to have impaired humoral immune responses and therefore a limited ability to clear SARS-CoV-2 without therapeutic intervention [7]. The combination of an RNA-dependent RNA polymerase inhibitor (e.g., remdesivir) and a 3CL protease inhibitor (e.g., ensitrelvir or nirmatrelvir) has been shown to enhance the clearance of SARS-CoV-2, facilitating a more rapid reduction in viral load and eventually lowering the risk of severe disease and mortality [19-23]. Additionally, in vitro studies have demonstrated that combining remdesivir with a 3CL protease inhibitor interferes with different stages of viral replication and maturation, exhibiting a potent antiviral effect [24]. Despite the limited reports regarding the use of ensitrelvir as a 3CL protease inhibitor in combination therapy [21], our findings suggest that the combination of remdesivir and ensitrelvir may be effective in promoting SARS-CoV-2 clearance in vivo.

The timing of antiviral therapy initiation is considered critical with respect to viral load reduction and improvement of clinical outcomes. Orth et al. demonstrated that initiating combination therapy within five days of symptom onset significantly reduced viral load; in contrast, treatment initiation after five days was associated with sustained high levels of viral RNA over time [11]. Although Choi et al. did not report viral load dynamics, they reported that initiating combination therapy within five days of symptom onset was associated with reduced mortality rate and prevention of disease progression [23]. In our study, the timing of post-treatment antigen testing was earlier in the combination therapy group than in the monotherapy group (median, seven days vs. 10 days). Despite this earlier measurement point, the combination therapy group demonstrated significantly lower post-treatment antigen levels. Moreover, the interval between symptom onset and treatment initiation was longer in the combination therapy group (median, 13 days vs. 0 days), yet lower mortality and a significant reduction in antigen levels were observed. These findings suggest that combination therapy may provide clinical benefit even when initiated beyond the conventional treatment window, particularly in immunocompromised patients with a history of anti-CD20 monoclonal antibody therapy, who may experience prolonged viral replication and therefore remain responsive to antiviral intervention later in the disease course.

This study has certain limitations. First, the cohort size was small (n = 17), and the number of fatal cases was limited (n = 9), which precluded robust multivariable analysis. Second, we did not perform imputation for missing data given the small cohort size and the possibility that the missing values were not completely random. Finally, the types of nucleic acid amplification tests and antigen quantification assays used were heterogeneous; furthermore, the timing and frequency of testing were not standardized. Survival curve interpretation is limited by the small cohort and unequal baseline disease severity. Because treatment assignment was not randomized and clinicians tended to administer combination therapy to patients with more severe disease, confounding by indication represents a major limitation that may influence the observed differences in mortality. This study included only 17 patients, which substantially limits statistical power and generalizability. As such, the findings should be considered hypothesis-generating rather than confirmatory. Therefore, our findings should be interpreted with caution, and further validation in larger prospective studies is warranted.

## Conclusions

In patients with a history of anti-CD20 monoclonal antibody therapy hospitalized with COVID-19, combination therapy with ensitrelvir and remdesivir was associated with lower long-term mortality and reduced post-treatment viral antigen levels compared with antiviral monotherapy, despite more severe baseline characteristics. However, given the small retrospective cohort and potential confounding by indication, these findings should be interpreted cautiously and considered hypothesis-generating. Larger prospective studies are required to validate these observations and to determine optimal treatment timing and duration.
